# MED-ChatGPT CoPilot: a ChatGPT medical assistant for case mining and adjunctive therapy

**DOI:** 10.3389/fmed.2024.1460553

**Published:** 2024-10-16

**Authors:** Wei Liu, Hongxing Kan, Yanfei Jiang, Yingbao Geng, Yiqi Nie, Mingguang Yang

**Affiliations:** ^1^School of Medical Information Engineering, Anhui University of Traditional Chinese Medicine, Hefei, Anhui, China; ^2^Anhui Computer Application Research Institute of Chinese Medicine, China Academy of Chinese Medical Sciences, Hefei, Anhui, China

**Keywords:** ChatGPT, large language model, data mining, prompt engineering, local knowledge base

## Abstract

**Background:**

The large-scale language model, GPT-4-1106-preview, supports text of up to 128 k characters, which has enhanced the capability of processing vast quantities of text. This model can perform efficient and accurate text data mining without the need for retraining, aided by prompt engineering.

**Method:**

The research approach includes prompt engineering and text vectorization processing. In this study, prompt engineering is applied to assist ChatGPT in text mining. Subsequently, the mined results are vectorized and incorporated into a local knowledge base. After cleansing 306 medical papers, data extraction was performed using ChatGPT. Following a validation and filtering process, 241 medical case data entries were obtained, leading to the construction of a local medical knowledge base. Additionally, drawing upon the Langchain framework and utilizing the local knowledge base in conjunction with ChatGPT, we successfully developed a fast and reliable chatbot. This chatbot is capable of providing recommended diagnostic and treatment information for various diseases.

**Results:**

The performance of the designed ChatGPT model, which was enhanced by data from the local knowledge base, exceeded that of the original model by 7.90% on a set of medical questions.

**Conclusion:**

ChatGPT, assisted by prompt engineering, demonstrates effective data mining capabilities for large-scale medical texts. In the future, we plan to incorporate a richer array of medical case data, expand the scale of the knowledge base, and enhance ChatGPT’s performance in the medical field.

## 1 Introduction

In November 2022, OpenAI released ChatGPT, a GPT-3.5 Turbo-powered intelligent chatbot, marking a significant advancement in knowledge management and artificial intelligence content creation (1). This technology has significantly enhanced the ability to comprehend and generate text, assisting researchers and medical experts in writing literature reviews and abstracts, as well as suggesting structures and references, and it can even be used to write draft papers (2). Leveraging its efficient semantic comprehension, language generation, and logical reasoning capabilities, ChatGPT has been widely applied in various fields such as media, education, healthcare, customer service, law, and data processing, garnering considerable attention (3, 4).

In the medical field, various studies (5–8) have explored the potential of ChatGPT, indicating that it can alleviate the burden on physicians without disrupting existing workflows. These studies emphasize the role of ChatGPT in enhancing the humane aspect of caregiving, including empathy and sensitivity to patients’ emotional needs, which current large language models (LLMs) struggle to fully achieve. Faced with the unequal distribution of global medical resources, where developed countries have access to advanced medical technology and well-trained healthcare professionals while developing countries often struggle with limited medical infrastructure and insufficient healthcare providers (9, 10), it becomes necessary to develop tools that can efficiently address common health issues. LLMs possess the capability to utilize knowledge from various disciplines to efficiently handle labor-intensive and time-consuming tasks, such as literature search, medical entity recognition, and data analysis (11–13). Recent studies have shown that trained models are capable of reformulating texts to convey empathy more effectively, which can enhance mental health treatments (14). For example, these models can rephrase clinical advice or supportive messages to make them more comforting and understanding for patients. Additionally, LLMs have demonstrated potential in various medical fields such as radiology, ophthalmology, medical insurance, and urology (15–18).

Despite this, extracting medical information from the plethora of literature and storing it in a local knowledge base remains a challenge. Traditional methods are labor-intensive, weak in generalization capabilities, and often require familiarity with specific software tools or computer programming skills (19). Additionally, the high complexity of medical information (20, 21) and issues such as bottlenecks in data annotation (22) represent significant challenges that cannot be overlooked. However, with the emergence of high-performance, LLMs that support up to 128 k context, such as GPT-4-1106-preview (23), this process is expected to be revolutionized. As a data processing assistant, ChatGPT can work collaboratively with human researchers to advance text mining and data analysis (24, 25). ChatGPT’s capabilities stem from its vast pre-trained text corpus, which is a large collection of diverse texts used during the model’s training (26). This enables ChatGPT to naturally excel in language comprehension and named-entity recognition, identifying professional terms such as disease and drug names without additional training (27). Moreover, ChatGPT is adept at identifying and associating abbreviations with their full forms in medical data mining, such as “RA” (Rheumatoid Arthritis), “LFTs” (Liver Function Tests), and “PTT” (Partial Thromboplastin Time). This ability is crucial for reducing the quantity of duplicate “unique entities” in datasets that arise due to the use of various abbreviations, helping to avoid redundant data without new information. In contrast, traditional natural language processing methods often fail to recognize abbreviations and full names without a manually compiled medical abbreviation dictionary (28).

With the continuous advancement of artificial intelligence technology, AI has played a key role in various subfields of medicine, such as pancreatic cancer, liver cancer, digestive system diseases, and retinal pathologies (29–32). Deep learning and advanced algorithms aid physicians in more accurate diagnosis and prediction of diseases (33, 34). Research has also explored the use of AI in radiopathomics (the integration of radiology and pathology data to improve diagnostic accuracy) and diabetology (35, 36).

Although LLMs such as ChatGPT have made significant advancements in the medical field, enhancing the efficiency and accuracy of healthcare services, current research often focuses on the intelligent diagnosis of specific diseases. This leaves a gap in the development of comprehensive intelligent systems that are broadly applicable to multiple conditions. Additionally, the complexity, diversity, and challenges associated with data annotation in medical information processing remain significant hurdles. Studies indicate that well-designed prompts and contextual settings can substantially reduce the likelihood of ChatGPT generating erroneous information (24, 37). This insight has guided our approach in designing text data mining strategies, ensuring maximum efficiency and accuracy in GPT outputs. To address these issues, our research aims to utilize the latest ChatGPT model, which supports extensive context, to deeply mine medical information and assist in data annotation. Subsequently, we will construct a medical knowledge base derived from the deeply mined data, enhancing the application performance of LLMs in the medical domain. Finally, we propose a system named “MED-ChatGPT CoPilot,” which integrates case mining with auxiliary treatment suggestions. This system is designed to provide medical professionals with an efficient and rapid method for case mining and data annotation, while also serving as a convenient medical knowledge advisor for patients.

## 2 Methods and experimental design

**Figure 1:**
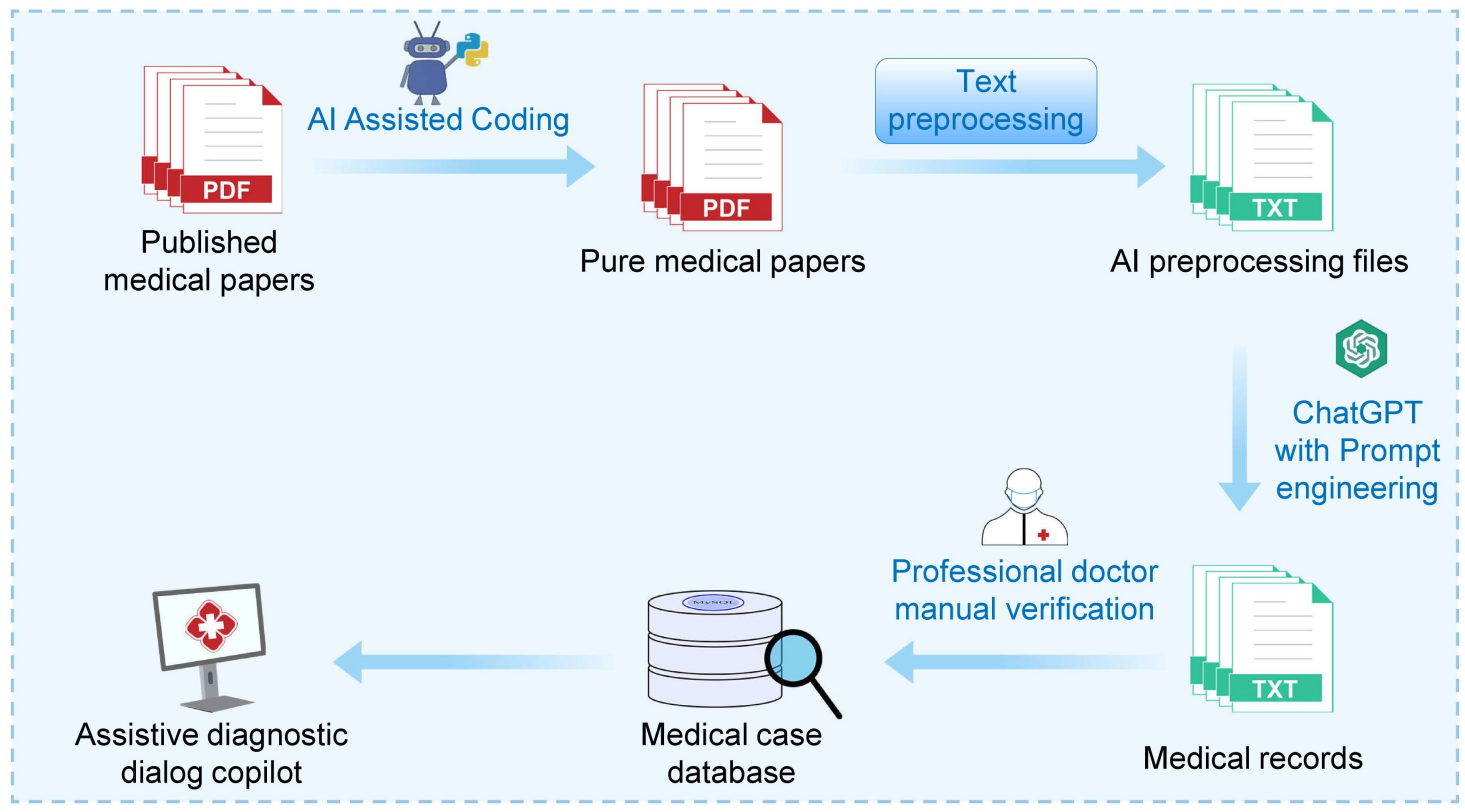
Schematic diagram of the MED-ChatGPT CoPilot workflow. The diagram illustrates a MED-ChatGPT CoPilot-assisted workflow that begins with “Published medical papers” being preprocessed using a Python script generated with ChatGPT’s assistance. The preprocessing step removes extraneous information, such as references, acknowledgments, and other non-essential sections, resulting in “Pure medical papers.” These pure medical papers focus solely on relevant medical content and are then converted into text files to ensure compatibility and ease of processing by ChatGPT. Subsequently, through collaborative efforts between ChatGPT and Prompt Engineering, “Medical records” are generated. These records undergo professional medical review, and 241 cases are vectorized and indexed to construct the “Medical Case Database,” a local repository of medical knowledge. Finally, the integration of this local vector knowledge database with ChatGPT fulfills the objective of this study: to create a medical assistant for case mining and aiding diagnosis and treatment. It is important to note that the fifth step of the workflow involves using ChatGPT to compose script code to assist with tasks, a process which is interwoven throughout the entire workflow and elaborated on in Section 2.5. The reason for converting the papers into text files is that ChatGPT processes text more efficiently and accurately than PDFs. Text files are easier to parse and manipulate programmatically, ensuring that the relevant medical information is seamlessly integrated into the workflow.

The workflow of this study is divided into five steps: (1) Data preprocessing; (2) Design of text mining using ChatGPT; (3) Building a local knowledge base and calculating similarity vectors; (4) Developing an auxiliary inquiry system based on the ChatGPT API; and (5) Utilizing ChatGPT to write script codes for assistance with tasks (Figure 1).

### 2.1 Data preprocessing

In the data preprocessing stage of this study, special attention was given to the security and reliability of the selected medical data. We conducted an extensive literature search using academic literature platforms such as ScienceDirect, Web of Science, and PubMed, with keywords including “clinical guidelines,” “treatment guidelines,” and “treatment strategies.” Building upon this, we established a set of stringent screening criteria. Initially, we excluded all non-English literature, case reports, reviews, forum articles, brief communications, and expert opinion pieces, as well as studies that were not peer-reviewed or had a sample size of less than 100, to ensure the scientific quality and statistical power of the selected papers. Furthermore, we eliminated studies that lacked a clear mechanism of disease treatment or verification of treatment efficacy. The literature that passed this meticulous screening process needed to provide detailed descriptions of treatment strategies for specific diseases, be based on large clinical trials or multicenter studies and have clear data collection and analysis methodologies, which ensures both the quality of data and the broad applicability of the research.

To further ensure the high quality of the selected studies, we conducted an additional quality assessment by evaluating the average citation count of each paper. Only those papers with an average citation count of five or more were included, indicating that these studies have been recognized and validated by the scientific community. After this two-round screening process, we ultimately selected 306 high-quality medical research papers published within the past 5 years that covered various disease domains. Through this rigorous selection process, we ensured the scientific validity and practical utility of the chosen papers, providing a solid data foundation for this study.

To address the issue of text length limitations inherent in LLMs for text processing, we have devised an innovative technical approach. This approach involves filtering paragraphs directly related to disease diagnosis and treatment from the complexities of the literature. We utilize meticulously crafted regular expressions to identify and automatically exclude non-essential information, such as reference citations and acknowledgments, based on their formatting patterns. To ensure the accuracy and relevance of the filtering process, a professional doctor reviewed the criteria and results to confirm that only non-essential information was removed. This step was crucial to maintain the integrity and context of the medical information in the filtered data. Additionally, the detailed process and specific implementation methods have been compiled in the supplementary materials, available in the Data Availability section along with the corresponding code and datasets. Considering that excessive text length can impact the efficiency and effectiveness of the model (38), we have eliminated overly verbose and medically irrelevant text segments post-filtering. By retaining only the essential medical content, we aim to enhance the model’s performance without compromising data reliability. Through this series of preprocessing steps, we have ensured that each selected medical literature includes at least one disease and its corresponding treatment method and that the token size and format comply with the input requirements of the ChatGPT-4-1106-preview model, namely, keeping the context length under 128 thousand tokens.

Considering both the context length limitations of ChatGPT and the need to maintain the text’s quality and relevance, we adopted a balanced approach to preprocessing. While we acknowledge that extensive preprocessing could potentially limit the generalizability of our findings, we also recognize the critical need for ChatGPT to process information that is accurate and dense, enabling more effective case mining and support for diagnosis and treatment. Thus, our preprocessing was carefully calibrated to eliminate clearly non-essential content while preserving the vital details essential for understanding disease diagnostics and therapeutic strategies. By doing so, we achieved a compromise between the need for preprocessing and preserving the integrity and wide applicability of our study to diverse medical literature.

### 2.2 Text data mining design based on ChatGPT

**Figure 2:**
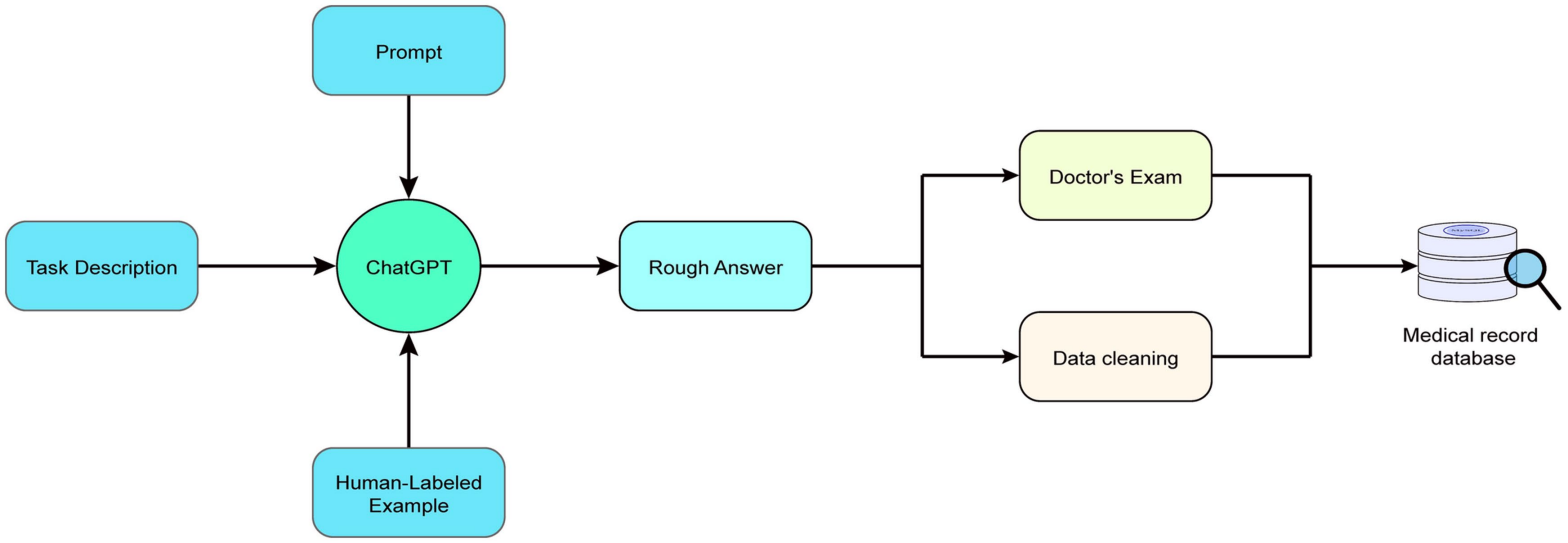
An overview of the workflow for medical text mining with ChatGPT assisted by prompt engineering. After the large model extracts preliminary data, the data undergo cleansing and verification by professional physicians before proceeding to the next step of building a local knowledge base, which will be detailed in Section 2.3.

Through repeated experimentation and fine-tuning, we determined the optimal prompt design and background setting (also known as task description) and appended cues after each question to enhance the efficiency and accuracy of GPT outputs and ensure the content’s standardization. Within the prompts, we required the large model to divide each disease-related information into six parts: (1) Disease name; (2) Clinical manifestations; (3) Recommended treatment protocols; (4) Recommended medications; (5) Precautions and side effects; and (6) Additional recommendations (such as lifestyle advice). The workflow diagram for this study’s ChatGPT-assisted medical text mining task using prompt engineering is presented in Figure 2. Specific prompts, background information, as well as input and output examples, can be found in Table 1.

**Table 1:** Detailed prompt and example documentation for study.

Task description	“context”: “You are a medical assistant specializing in answering common disease questions, answering questions based on the papers you have read. Use the provided context to answer the question. If the question is irrelevant in the context, or the context does not mention anything related to the disease, answer ‘Based on the information in the medical literature I have read so far, I cannot provide a reliable answer to this question. Please modify your question.”
Prompt	Please extract the following information from the provided text: Disease name, Clinical manifestations, Recommended treatment plan, Recommended medication, Precautions and side effects, Other suggestions (such as lifestyle advice). If the text contains information regarding other diseases, please also present it in the required format. If certain attributes of the disease are not mentioned, then there is no need to provide me with information about that disease. Then, refine the extracted information in the following format: Disease name:\n Clinical manifestations:\n Recommended treatment plan:\n Recommended medication:\n Precautions and side effects:\n Other suggestions:\n Please note that the recommended medication and its corresponding precautions and side effects should be matched correctly。If the requested information is not extracted, let me know so. Below is the text context to be extracted:
Input	“158. INFECTIONS DUE TO CAMPYLOBACTER AND RELATED SPECIES – Martin J. Blaser DEFINITION Bacteria of the genus Campylobacter and of the related genera Arcobacter and Helicobacter (Chap. 154) cause a variety of pyogenic infections. Although acute diarrheal illnesses are most common, these organisms may cause infections in virtually all parts of the body, especially in compromised hosts, and these infections may have late nonsuppurative sequelae. The designation Campylobacter comes from the Greek for “curved rod” and refers to the organism’s vibrio-like morphology.” … omission of the following text, which in total contains 2,276 words.
Output	Disease name: Campylobacter and related species infections Clinical manifestations: Diarrhea, abdominal pain, fever, Diarrheal illness may be grossly bloody, Cramping, pseudoappendicitis, Convulsions in febrile young children, Intermittent diarrhea or nonspecific abdominal pain without localizing signs (*C. fetus*),Prolonged relapsing systemic illness with fever, chills, and myalgias (*C. fetus*),Recurrent cellulitis with fever and bacteremia in immunocompromised hosts (*H. cinaedi*) Recommended treatment plan: Fluid and electrolyte replacement for diarrheal illnesses, Antimicrobial therapy for high fever, bloody diarrhea, severe diarrhea, persistence for more than 1 week, and worsening of symptoms, For systemic infections, empirical treatment with gentamicin, imipenem, or chloramphenicol, followed by susceptibility testing, Prolonged therapy for immunocompromised patients or endovascular infections Recommended medication: Erythromycin (250 mg orally four times daily or 30 to 50 mg/kg per day for children),Clarithromycin and azithromycin as alternatives, Ciprofloxacin (500 mg orally twice daily) or another fluoroquinolone for adults, Tetracycline and furazolidone as other alternatives, Gentamicin, imipenem, or chloramphenicol for systemic infections, Ciprofloxacin and amoxicillin/clavulanate for susceptible strains Precautions and side effects: Resistance to fluoroquinolones is increasing, Antimotility agents may prolong symptoms and are not recommended, Side effects specific to each medication should be considered and monitored Other suggestions: Cook poultry sufficiently to prevent infection, Avoid raw (unpasteurized) milk or untreated water, Practice good hygiene when in contact with household pets, Seek medical attention if symptoms persist or worsen

This table documents the specific prompts, background information, and examples of input and output used in detail in this study.

Moreover, OpenAI’s API allows researchers to precisely control key parameters of the GPT model, including “temperature” and “history.” The “temperature” parameter dictates the degree of conservatism or innovation in the model’s output, with values ranging from 0 (very conservative) to 1 (highly innovative). In this study, we set eleven different “temperature” values at intervals of 0.1, from 0 to 1, to test their effect on data mining efficacy. To ensure consistent experimental conditions, we conducted three independent rounds of ChatGPT tests for each “temperature” setting and recorded the outputs. Moreover, to eliminate the influence of “history” on the model’s outputs, we reset ChatGPT’s “history” parameter after each interaction, ensuring that each invocation started from a state with no memory, to avoid any impact from previous interactions on subsequent outputs. By comparing model performance under various settings, we found that the GPT-4-1106-preview model performed best with the “temperature” set to 0.1. Hence, we selected this parameter configuration as the standard setup for extracting medical text information.

### 2.3 Building a local knowledge base and vectorized similarity computation

Since the advent of ChatGPT, Large LLMs have been warmly received globally, and tool frameworks like LangChain (39) have been developed. LangChain is a framework designed specifically for developing applications based on LLMs, offering developers a range of modules and tools to simplify the integration process with LLMs. Using LangChain, developers can easily leverage language models to perform complex tasks, including text-to-image conversion, document question answering, and chatbot construction. In this research, we adopt the LangChain framework to deeply integrate pre-trained language models with a local knowledge base. Using “disease names” and “clinical manifestations” from medical case information to build an index, we then employ the text2vec-large-Chinese vectorization model (40) to vectorize the medical case data. This model not only demonstrates excellent vectorization capabilities for Chinese and English texts but is also fully open-source and free, aligning with our future plans to incorporate Chinese medical cases into the database.

While exploring different retrieval enhancement methods, we also considered other forms, such as the direct embedding of source files (41). However, we opted for vectorized similarity calculation and the construction of a local knowledge base because this approach provides different benefits for researchers and patients. For researchers, it offers a scalable and interpretable representation of medical case information, enabling complex semantic retrieval and deeper insights, which surpass simple text matching. On the other hand, for patients, our method emphasizes an intuitive and easy-to-manage user interface that makes medical information more accessible and understandable. Compared to file embedding, our approach focuses on the specific needs of each user group, ensuring that researchers can access detailed and nuanced data while patients receive clear and straightforward information. Furthermore, given the sensitivity and complexity of medical information, we believe that providing a clear, interpretable knowledge representation is more important than relying solely on automated, potentially difficult-to-explain embedding vectors.

After vectorizing the medical case data, the storage and retrieval of vector content rely on the Qdrant vector search database (42). Vector databases are an emerging means of data interaction that combine with abstract data representations produced by machine learning models, such as deep learning. Vector databases exhibit exceptional performance in applications such as semantic search and recommendation systems (43). Qdrant is an open-source vector database designed for the new generation of AI applications, using a cloud-native architecture and offering RESTful and gRPC APIs for embedding vector management. It supports search functions for images, voice, and video and can be integrated with AI engines, enhancing the breadth and depth of its applications. Additionally, Qdrant uses the Cosine Similarity algorithm to improve retrieval accuracy, the formula of which is as follows:


$$\mathrm{Similarity}\;\left(A,B\right)=\frac{A\cdot B}{A\times B}=\frac{\sum_{i+1}^{n}\left(A_{i}\times B_{i}\right)}{\sqrt{\sum_{i+1}^{n}A_{i}^{2}}\times \sqrt{\sum_{i=1}^{n}B_{i}^{2}}}\#$$

### 2.4 Establishing a ChatGPT-based auxiliary diagnostic chatbot

**Figure 3:**
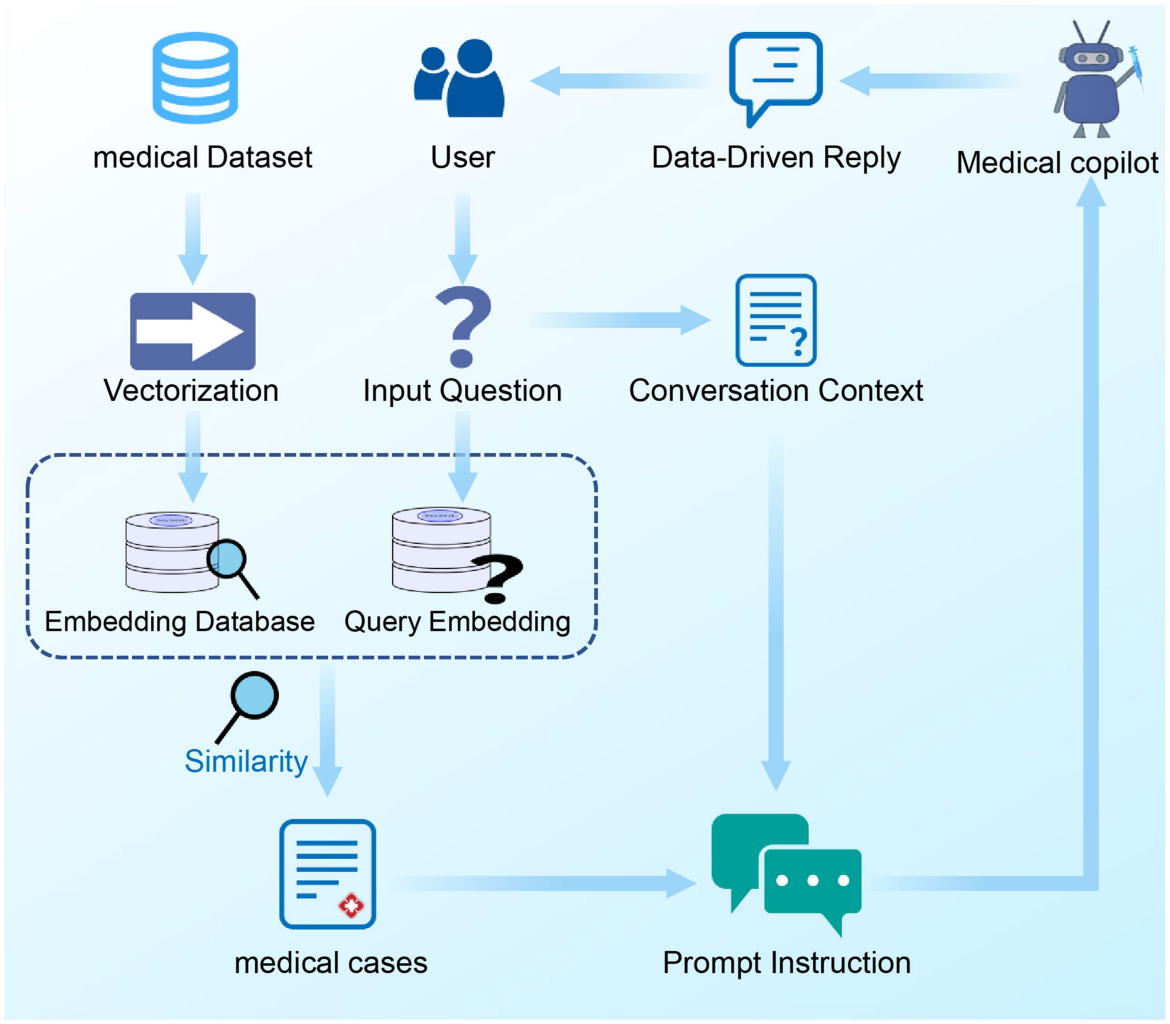
The integrated workflow of the medical chatbot. The integrated workflow of the medical chatbot. This workflow begins with the user initiating a query, which is then vectorized using a pre-trained language model. The system calculates the cosine similarity between the user’s query vector and vectors in the local medical case database. The top three matching medical cases are retrieved, and further matched with the user’s dialogue context to select the most relevant case. This selected case, combined with prompt engineering, is then input into the ChatGPT model. The ChatGPT model generates a response that is presented to the user through the interface.

After completing the vectorization embedding of medical case texts and constructing a corresponding local medical case vector database, this research utilized Python’s Streamlit package to build a front-end interface, enabling auxiliary diagnostic dialogues with users. When a user initiates a query, the system first processes the content of the query into a vectorized form. By calculating the cosine similarity between the user’s query vector and each vector in the medical case vector database, the system can precisely retrieve the text units that best match the user’s needs. After the optimal matching text unit is selected, it is applied in conjunction with the prompt engineering to the ChatGPT model. Benefiting from ChatGPT’s powerful capabilities in text generation and logical reasoning, this method combines the data-driven characteristics of the vector database, effectively enhancing the accuracy of the consultation and significantly reducing the tendency for “hallucination” phenomena that large-scale language models are prone to (44, 45). Ultimately, the system presents the processed results on the user interface. A schematic of the integrated workflow for the medical dialogue robot is presented in Figure 3.

To ensure the safety and accuracy of medical advice, this system’s chatbot processes queries solely from the database, thereby reducing the production of misleading information. Furthermore, the chatbot is capable of “remembering” previous conversations, including recognized contexts and pertinent medical case information, to maintain the coherence and data-driven nature of the responses.

### 2.5 ChatGPT-assisted script code generation work

In this study, we have fully leveraged the automation capabilities of ChatGPT to generate Python scripts that are used for parsing medical literature, generating prompts, processing text, and conducting data mining, with the results being output in a predefined format. Traditionally, this process required complex programming skills and a significant investment of time; however, with the efficient code generation capabilities of ChatGPT, this process has been significantly simplified and accelerated. For instance, in less than a second, the GPT model is capable of generating a data preprocessing script, which can automatically convert PDF-formatted papers into clean medical text files devoid of irrelevant information, markedly reducing the workload and time costs. Researchers simply need to describe their requirements and the desired output format in natural language, and ChatGPT rapidly generates the corresponding Python code. This code can be directly copied, pasted, modified, and executed, greatly enhancing research efficiency. Should any errors arise during the execution of the code, this large-scale model also provides instant assistance to help researchers quickly identify and correct these errors. We have thoroughly validated and tested the generated scripts to ensure their accuracy and reliability in parsing and processing medical texts. Furthermore, to promote reusability, transparency, and assessment of this study, these scripts (including pdf_to_pool.py, remove_ref.py, txt_to_csv.py, etc.) have been shared in the supplementary materials. This section has been expressly added to highlight the possibility that even non-programmers, with the assistance of GPT, can complete and fine-tune automated workflows.

### 2.6 Evaluation of experimental design

In order to investigate the impact of the MED-ChatGPT medical assistant on the time efficiency of medical case mining and its quality of responding to a specific set of medical questions after being augmented with a medical knowledge base, this study designed a series of evaluative experiments.

To assess the efficiency of ChatGPT in mining medical texts, we devised a repeated comparative validation experiment. Initially, we compiled a pool of 306 high-quality medical research papers published within the last 5 years. From this pool, we randomly selected 100 papers to ensure a representative sample for our study. The random selection was performed using Python’s `random` module, specifically the `sample` function, which ensures each paper has an equal probability of being chosen. For each experimental round, 20 papers were chosen for comparative validation, with a total of five rounds conducted. In each round, we maintained constant other variables (such as API interface parameters, prompts, and temperature) and assigned both the ChatGPT-4-1106-preview model and three Chinese medical professionals with master’s degrees and over 5 years of clinical experience to process these papers. We recorded the time taken by each to complete the tasks in order to calculate the average time. To account for potential fatigue phenomena human participants might experience when processing large volumes of text, we spaced the experiment over 5 days, with 1 day between each round.

Subsequently, to evaluate the performance of MED-ChatGPT CoPilot in medical question-answering tasks, we designed a series of experiments aimed at comparing the question-answering quality of the ChatGPT model, enhanced with a medical knowledge base, against the original baseline model when addressing a specific set of medical questions. The experiments utilized the MultiMedQA medical benchmark dataset (46), which comprises questions that include multiple-choice and long-form responses. From its subset, the MedQA dataset (47), we randomly selected 300 questions as our test samples, ensuring the generalizability and randomness of the test results. The reason for using only a subset of the MedQA dataset, rather than the complete benchmark dataset, is that these questions all followed the style guidelines of the United States Medical Licensing Examination, consisting solely of single-choice questions. This approach was chosen to reduce subjectivity in the evaluation and ensure the definitiveness of the answers. We compared the performance of the original baseline model (ChatGPT-4-1106-preview) with that of the model enhanced with a medical knowledge base, using the same set of questions for both models, and recorded all question-answer pairs (see Supplementary Data). The evaluation criterion was based on the proportion of questions correctly answered by the models relative to the total number of test questions, thereby measuring and comparing the performance of the two models.

## 3 Results

### 3.1 Text mining time performance evaluation

**Figure 4:**
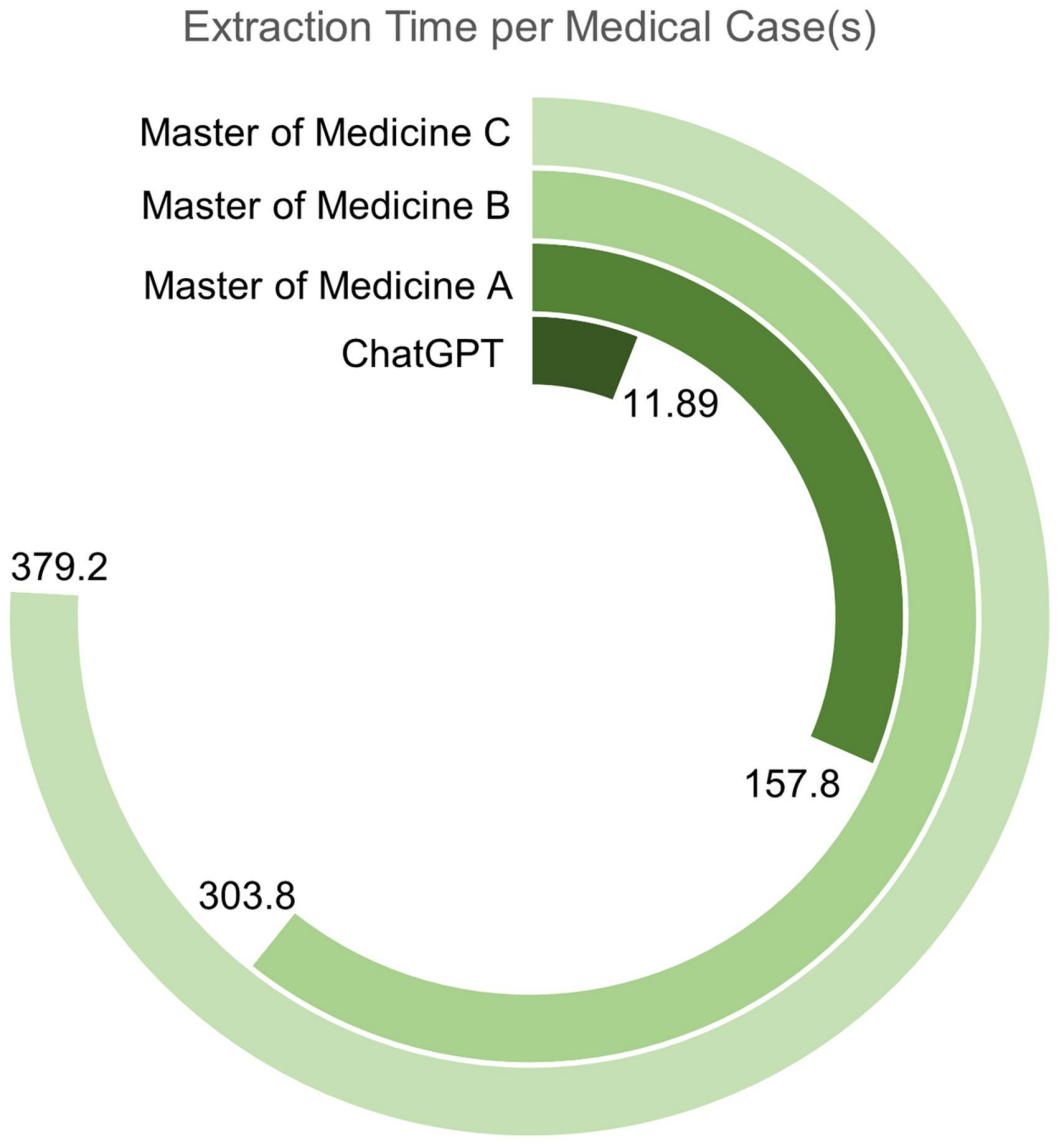
The timeline comparison chart for medical text information mining in this study. It records the average time required for three medical masters and the medical assistant from this study to extract information from the same medical case, clearly demonstrating their relative efficiency in completing this task.

In the domain of medical text mining efficiency, experimental results (Figure 4) demonstrate that the efficiency of information extraction from medical cases by large-scale models significantly surpasses that of a human with substantial medical knowledge. In the comparative study of medical text mining efficiency, we calculated the mean, standard deviation, and 95% confidence interval for the time taken by the GPT model and a human with a master’s degree in medicine to process literature. The GPT model requires an average of only 11.89 s per document (with a standard deviation of 1.76 s and a 95% confidence interval ranging from 10.96 to 12.82 s), totaling less than 4 min to process 20 documents. In contrast, the average time taken by the medical master’s degree holder to process the same number of documents ranges from 157.8 to 379.2 s per document (with standard deviations of 21.58 s and 46.28 s, and 95% confidence intervals of 150.34–165.26 s and 359.14–399.26 s, respectively), and this duration already takes into account the assistance of English translation software. This further highlights the advantages of GPT in multilingual processing.

### 3.2 Evaluation of the ChatGPT model enhanced with a local knowledge base for medical knowledge performance

In the assessment of medical knowledge performance, this study compared two models utilizing identical internal interface parameters: the original ChatGPT-4-1106-preview base model and MED-ChatGPT CoPilot, the model developed in this study augmented with a medical knowledge base. The evaluation results are presented in Table 2.

**Table 2:** Performance statistics of MED-ChatGPT CoPilot versus the original model on a medical knowledge question set.

Model name	Correct answers	Incorrect answers	Total accuracy (%)	Improvement ratio (%)
ChatGPT-4-1106-preview	215	85	71.67	7.90
MED-ChatGPT CoPilot	232	68	77.33	

After analyzing 300 randomly selected sample questions, the initial ChatGPT-4-1106-preview baseline model exhibited an accuracy rate of 71.67%, whereas MED-ChatGPT CoPilot demonstrated an accuracy rate of 77.33%. This comparative result clearly indicates that, on a specific set of medical questions, MED-ChatGPT CoPilot surpassed the original baseline model, with an increased accuracy of 7.90%.

However, it must be noted that the performance improvement is, to some extent, constrained by the current scale of the knowledge base. Given that the knowledge base presently contains only 241 medical case entries, the observed enhancement in performance is already significant. It is reasonable to anticipate that with further expansion of the knowledge base, the model’s performance could see even greater improvements.

### 3.3 Consulting diagnostic and therapeutic recommendations and medication suggestions through an auxiliary robotic system

**Figure 5:**
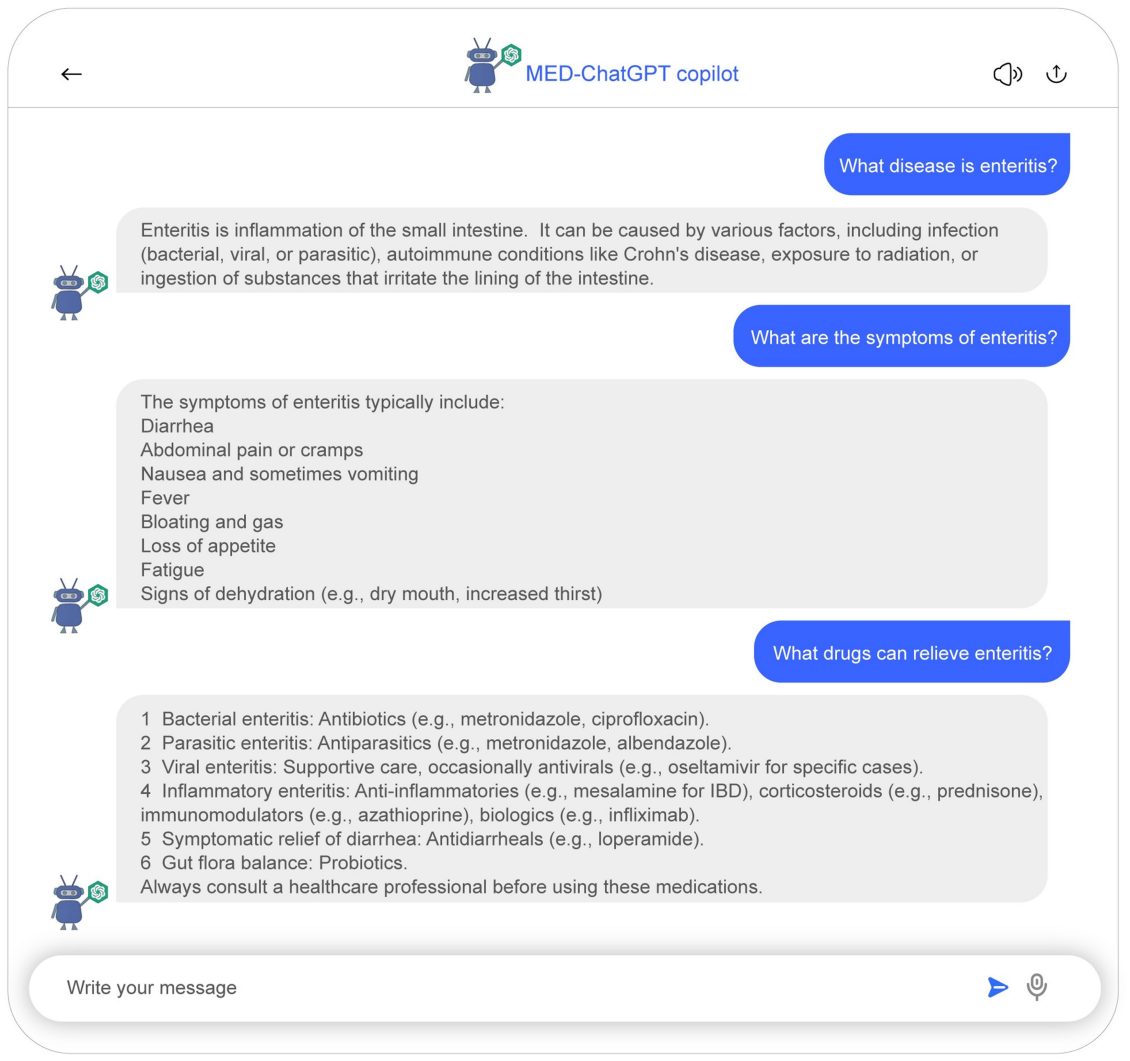
Demonstrative sample of the medical chatbot interaction. Users are able to inquire about detailed information regarding diseases, which encompasses an introduction to the disease, clinical manifestations, as well as recommendations for medication.

The developed system maximizes interactivity and user experience by converting database information into a conversational format, as detailed in Figure 3. This medical dialogue bot facilitates the acceleration of research findings to clinical application. Built upon ChatGPT’s medical assistant architecture, it supports medical case mining and diagnostic support. All conversational data are derived from detailed analyses of medical papers, ensuring safety and reliability. Figure 5 presents a schematic representation of the MED-ChatGPT copilot interacting with a user for medical consultation. In this dialogue display, the user can inquire about detailed information regarding a disease, encompassing key information such as clinical manifestations and medication recommendations.

In summary, the results indicate that the MED-ChatGPT CoPilot model, enhanced with a specifically curated local medical knowledge base, shows notable improvements in medical text mining and diagnostic assistance. This model provides high-quality, peer-reviewed medical recommendations, ensuring higher relevance and accuracy in the medical field. The MED-ChatGPT CoPilot-supported workflow excels in mining time efficiency and annotation efficiency, significantly simplifying medical research, case mining, and text annotation tasks.

## 4 Discussion

### 4.1 Background of existing research

Medical text mining aims to extract valuable information from complex medical data to aid in diagnosis, treatment, and disease prediction. Previous studies have applied various machine learning algorithms, including k-nearest neighbors, decision trees, logistic regression, naive Bayes, and support vector machines, to this task (48–50). However, these efforts face significant challenges. International variations in medical information and the scarcity of annotated databases hinder the effectiveness of medical data mining.

Traditional methods rely heavily on keyword matching, which often leads to suboptimal outcomes. The advent of Electronic Health Records (EHRs) has improved data standardization, yet it introduces new concerns regarding patient privacy and the legal acquisition of EHR data (51, 52). Despite advancements, these systems still grapple with non-standardized terminology and fragmented information distribution (53).

Additionally, the COVID-19 pandemic has sparked interest in integrating artificial intelligence into medical assistance systems. Numerous projects have attempted to incorporate AI into disease diagnosis and treatment (54–57), but they mostly still rely on traditional methods, such as keyword matching approaches. Recent innovations include multimodal approaches, such as combining neuroimaging and voice analysis to diagnose Parkinson’s disease (58), highlighting the potential for more complex systems. However, these advancements are still in progress, and there is an urgent need for more comprehensive, accurate, and personalized AI-driven medical tools.

### 4.2 Key findings and innovations

Our study demonstrates that ChatGPT can significantly enhance efficiency and accuracy in medical text mining and diagnostic support. By integrating a local medical knowledge base with vectorized similarity computations, we improved the precision of retrieving relevant medical cases and ensured user-friendly data presentation. The MED-ChatGPT CoPilot model, combining ChatGPT with curated medical data, notably increased accuracy in medical question-answering tasks from 71.67 to 77.33%. Additionally, the use of ChatGPT for automated script generation streamlined the research process, making advanced medical text processing more accessible. These innovations collectively advance the field of medical text mining and diagnostic assistance.

### 4.3 Identified limitations and challenges

Despite the significant advantages demonstrated by the MED-ChatGPT CoPilot model in medical text mining and diagnostic assistance, several limitations and challenges were identified in this study. One major challenge is the inherent dependency on the quality and comprehensiveness of the local medical knowledge base. Although the curated database of 241 medical cases provided a solid foundation for enhancing the model’s accuracy, its relatively limited scope means it may not cover all possible medical conditions or the latest research developments comprehensively. This constraint could lead to gaps in the model’s diagnostic capabilities, especially for rare or newly emerging diseases.

Another area warranting careful consideration is the model’s ability to provide medical advice. Although MED-ChatGPT CoPilot has shown to outperform standard search engines such as Google by providing more structured and comprehensive diagnostic suggestions, there are instances where the recommendations from the model appear similar to those generated by general search engines. For example, while a Google search for symptoms of enteritis might list potential causes such as bacterial, viral, or parasitic infections, our model goes further by suggesting specific tests based on patient symptoms and history to differentiate these causes. However, this enhancement is sometimes subtle, and the perceived similarity in the output can undermine the perceived value of using our specialized system over a general search engine.

The model’s performance enhancement observed in this study is also influenced by the specific configuration and tuning parameters, such as the temperature setting, which were determined through iterative experimentation. While these settings provided optimal results for our dataset, they might not be universally applicable across different medical domains or datasets, necessitating further fine-tuning and validation for broader applicability.

Moreover, while this study did not utilize any personal or private patient data, incorporating detailed individual patient data could further enhance the system’s capability in assisting medical diagnoses and treatments. In the future, we aim to integrate legally compliant and secure patient records to enrich the system. Ensuring the reliability and legality of data handling will be paramount throughout this process to safeguard patient privacy and comply with data protection regulations.

### 4.4 Future research directions and hypotheses

In light of the findings and challenges identified in this study, several future research directions and hypotheses have emerged. One critical area for future work is the expansion and enrichment of the local medical knowledge base. Increasing the number of medical cases and integrating the latest research developments will likely enhance the model’s diagnostic capabilities, particularly for rare and emerging diseases. This expansion could be achieved through continuous updates and collaboration with medical institutions to incorporate new clinical data and treatment protocols.

Another promising direction is the incorporation of personalized patient data into the system, which could significantly improve the relevance and accuracy of diagnostic and therapeutic recommendations. However, this approach necessitates stringent measures to ensure data privacy and compliance with legal regulations, such as the General Data Protection Regulation (GDPR). Developing robust data anonymization techniques and secure data handling protocols will be crucial in this endeavor.

The integration of multimodal data sources, such as imaging and genomic data, with text-based information presents another exciting prospect. Combining these diverse data types could provide a more comprehensive understanding of complex diseases, leading to more accurate diagnoses and personalized treatment plans. Exploring advanced machine learning techniques, such as multimodal learning, could facilitate this integration.

By pursuing these research directions, we aim to further refine and expand the capabilities of our MED-ChatGPT CoPilot, ultimately contributing to more effective and personalized medical care.

## 5 Conclusion

The MED-ChatGPT copilot effectively utilizes prompt engineering techniques and a local knowledge base to construct a high-precision, reliable medical assistant, providing an innovative and efficient solution for medical text mining and adjunctive diagnosis.

## Data Availability

The datasets presented in this study can be found in online repositories. The names of the repository/repositories and accession number(s) can be found in the article/supplementary material.
